# Supplementary material for: Unveiling the Bioactive Potential of the Invasive Jellyfish Phyllorhiza punctata Through Integrative Transcriptomic and Proteomic Analyses
Source: Biomolecules. 2025 Aug 4;15(8):1121. doi: 10.3390/biom15081121 (PMC12383608; doi:10.3390/biom15081121)
Supplement: Supplementary file 1 [file biomolecules-15-01121-s001.zip › Suplementary Material/FileS3/PEPFOLD4/XP_065053553_4355/clusters.html]

 PEP-FOLD clusters


Download archive of all models  here   

|  |  |  |
| --- | --- | --- |
| # | file | sOPEP |
| # cluster 1 - Size: 27 |  |  |
|  | XP\_065053553\_4355-model1 | -9.25508 |
|  | XP\_065053553\_4355-model1.1 | -9.25508 |
|  | XP\_065053553\_4355-model1.2 | -9.25508 |
|  | XP\_065053553\_4355-model1.3 | -9.25508 |
|  | XP\_065053553\_4355-model1.4 | -9.25508 |
|  | XP\_065053553\_4355-model1.5 | -9.25508 |
|  | XP\_065053553\_4355-model1.6 | -9.25508 |
|  | XP\_065053553\_4355-model1.7 | -9.25508 |
|  | XP\_065053553\_4355-model1.8 | -9.25508 |
|  | XP\_065053553\_4355-model1.9 | -9.25508 |
|  | XP\_065053553\_4355-model1.10 | -9.25508 |
|  | XP\_065053553\_4355-model1.11 | -9.25508 |
|  | XP\_065053553\_4355-model1.12 | -9.25508 |
|  | XP\_065053553\_4355-model1.13 | -9.25508 |
|  | XP\_065053553\_4355-model1.14 | -9.25508 |
|  | XP\_065053553\_4355-model1.15 | -9.25508 |
|  | XP\_065053553\_4355-model1.16 | -9.25508 |
|  | XP\_065053553\_4355-model1.17 | -9.25508 |
|  | XP\_065053553\_4355-model1.18 | -9.25508 |
|  | XP\_065053553\_4355-model1.19 | -9.20185 |
|  | XP\_065053553\_4355-model1.20 | -9.13921 |
|  | XP\_065053553\_4355-model1.21 | -9.13921 |
|  | XP\_065053553\_4355-model1.22 | -9.13921 |
|  | XP\_065053553\_4355-model1.23 | -9.13567 |
|  | XP\_065053553\_4355-model1.24 | -9.08254 |
|  | XP\_065053553\_4355-model1.25 | -9.03475 |
|  | XP\_065053553\_4355-model1.26 | -8.91041 |
| # cluster 2 - Size: 73 |  |  |
|  | XP\_065053553\_4355-model2 | -6.94784 |
|  | XP\_065053553\_4355-model2.1 | -6.94784 |
|  | XP\_065053553\_4355-model2.2 | -6.94784 |
|  | XP\_065053553\_4355-model2.3 | -6.94784 |
|  | XP\_065053553\_4355-model2.4 | -6.94784 |
|  | XP\_065053553\_4355-model2.5 | -6.92301 |
|  | XP\_065053553\_4355-model2.6 | -6.92301 |
|  | XP\_065053553\_4355-model2.7 | -6.91422 |
|  | XP\_065053553\_4355-model2.8 | -6.91422 |
|  | XP\_065053553\_4355-model2.9 | -6.90777 |
|  | XP\_065053553\_4355-model2.10 | -6.89967 |
|  | XP\_065053553\_4355-model2.11 | -6.89445 |
|  | XP\_065053553\_4355-model2.12 | -6.89445 |
|  | XP\_065053553\_4355-model2.13 | -6.89445 |
|  | XP\_065053553\_4355-model2.14 | -6.89445 |
|  | XP\_065053553\_4355-model2.15 | -6.89445 |
|  | XP\_065053553\_4355-model2.16 | -6.85161 |
|  | XP\_065053553\_4355-model2.17 | -6.85161 |
|  | XP\_065053553\_4355-model2.18 | -6.85161 |
|  | XP\_065053553\_4355-model2.19 | -6.85161 |
|  | XP\_065053553\_4355-model2.20 | -6.81727 |
|  | XP\_065053553\_4355-model2.21 | -6.80673 |
|  | XP\_065053553\_4355-model2.22 | -6.80673 |
|  | XP\_065053553\_4355-model2.23 | -6.80673 |
|  | XP\_065053553\_4355-model2.24 | -6.80673 |
|  | XP\_065053553\_4355-model2.25 | -6.80673 |
|  | XP\_065053553\_4355-model2.26 | -6.80673 |
|  | XP\_065053553\_4355-model2.27 | -6.80673 |
|  | XP\_065053553\_4355-model2.28 | -6.80673 |
|  | XP\_065053553\_4355-model2.29 | -6.80673 |
|  | XP\_065053553\_4355-model2.30 | -6.80673 |
|  | XP\_065053553\_4355-model2.31 | -6.80673 |
|  | XP\_065053553\_4355-model2.32 | -6.80673 |
|  | XP\_065053553\_4355-model2.33 | -6.80673 |
|  | XP\_065053553\_4355-model2.34 | -6.80673 |
|  | XP\_065053553\_4355-model2.35 | -6.797 |
|  | XP\_065053553\_4355-model2.36 | -6.797 |
|  | XP\_065053553\_4355-model2.37 | -6.797 |
|  | XP\_065053553\_4355-model2.38 | -6.79589 |
|  | XP\_065053553\_4355-model2.39 | -6.77717 |
|  | XP\_065053553\_4355-model2.40 | -6.77086 |
|  | XP\_065053553\_4355-model2.41 | -6.7588 |
|  | XP\_065053553\_4355-model2.42 | -6.74574 |
|  | XP\_065053553\_4355-model2.43 | -6.74574 |
|  | XP\_065053553\_4355-model2.44 | -6.72814 |
|  | XP\_065053553\_4355-model2.45 | -6.72814 |
|  | XP\_065053553\_4355-model2.46 | -6.72814 |
|  | XP\_065053553\_4355-model2.47 | -6.72814 |
|  | XP\_065053553\_4355-model2.48 | -6.72814 |
|  | XP\_065053553\_4355-model2.49 | -6.72525 |
|  | XP\_065053553\_4355-model2.50 | -6.7204 |
|  | XP\_065053553\_4355-model2.51 | -6.7204 |
|  | XP\_065053553\_4355-model2.52 | -6.70897 |
|  | XP\_065053553\_4355-model2.53 | -6.70106 |
|  | XP\_065053553\_4355-model2.54 | -6.70106 |
|  | XP\_065053553\_4355-model2.55 | -6.69935 |
|  | XP\_065053553\_4355-model2.56 | -6.69287 |
|  | XP\_065053553\_4355-model2.57 | -6.68621 |
|  | XP\_065053553\_4355-model2.58 | -6.68621 |
|  | XP\_065053553\_4355-model2.59 | -6.68621 |
|  | XP\_065053553\_4355-model2.60 | -6.68621 |
|  | XP\_065053553\_4355-model2.61 | -6.68621 |
|  | XP\_065053553\_4355-model2.62 | -6.66758 |
|  | XP\_065053553\_4355-model2.63 | -6.64353 |
|  | XP\_065053553\_4355-model2.64 | -6.64353 |
|  | XP\_065053553\_4355-model2.65 | -6.64353 |
|  | XP\_065053553\_4355-model2.66 | -6.63704 |
|  | XP\_065053553\_4355-model2.67 | -6.62366 |
|  | XP\_065053553\_4355-model2.68 | -6.62366 |
|  | XP\_065053553\_4355-model2.69 | -6.61752 |
|  | XP\_065053553\_4355-model2.70 | -6.5627 |
|  | XP\_065053553\_4355-model2.71 | -6.53302 |
|  | XP\_065053553\_4355-model2.72 | -6.45287 |
|  |  |  |
